# Implications for the diagnosis of aspiration and aspergillosis in critically ill patients with detection of galactomannan in broncho-alveolar lavage fluids

**DOI:** 10.1038/s41598-025-85644-5

**Published:** 2025-01-15

**Authors:** Simon Dubler, Michael Etringer, Christoph Lichtenstern, Thorsten Brenner, Stefan Zimmermann, Paul Schnitzler, Bettina Budeus, Fabian Rengier, Paulina Kalinowska, Yuan Lih Hoo, Markus A. Weigand

**Affiliations:** 1 Department of Anesthesiology, Medical Faculty, Heidelberg University, 69120 Heidelberg, Germany; 2 Department of Anaesthesiology and Intensive Care Medicine, University Hospital Essen, University Duisburg- Essen, 45147 Essen, Germany; 3 Division Bacteriology, Department of Infectious Diseases, Medical Microbiology and Hygiene, Heidelberg University Hospital, 69120 Heidelberg, Germany; 4 Department of Infectious Diseases, Virology, Heidelberg University Hospital, 69120 Heidelberg, Germany; 5 Institute of Cell Biology (Cancer Research), University of Duisburg-Essen, 45147 Essen, Germany; 6 Clinic for Diagnostic and Interventional Radiology, Heidelberg University Hospital, 69120 Heidelberg, Germany; 7 Translational Lung Research Centre Heidelberg (TLRC), Member of the German Centre for Lung Research (DZL), University of Heidelberg, 69120 Heidelberg, Germany

**Keywords:** Aspergillosis, *Aspergillus* spp., Invasive pulmonary aspergillosis, Critical illness, Intensive care unit, Pneumonia, Fungal infection, Diagnostic markers, Translational research

## Abstract

Invasive infections with *Aspergillus fumigatus* in ICU patients are linked to high morbidity and mortality. Diagnosing invasive pulmonary aspergillosis (IPA) in non-immunosuppressed patients is difficult, as *Aspergillus* antigen (galactomannan [GM]) may have other causes. This retrospective study analyzed 160 ICU surgical patients with positive GM in broncho-alveolar lavage fluid (BALF), classifying them based on AspICU criteria for suspected IPA (pIPA) or aspiration. Patients with pIPA had higher disease severity than those with aspiration, including higher dialysis rates, organ transplantation, corticosteroid use, and Sequential Organ Failure Assessment (SOFA) score. Aspergillus culture was positive in 47.0% of pIPA cases but only 2.6% of aspiration cases (*p* < 0.001). SOFA score at first positive GM in BALF independently predicted 28-day mortality. In surgical patients with a positive GM in BALF, aspiration is more likely if there’s no corticosteroid therapy, negative Aspergillus culture, and a history of aspiration events. Diagnosis of pIPA requires Aspergillus culture or prior corticosteroid therapy in this cohort of critically ill patients.

**Supplementary Information:**

The online version contains supplementary material available at 10.1038/s41598-025-85644-5.

## Introduction

Invasive fungal diseases (IFD) have become a major threat in critically ill patients as the population at risk continues to rise^1–3^. Classical (host) risk factors for IFD, including neutropenia due to chemotherapy and allogeneic stem cell transplantation (HSCT), have been known for years^4^. New groups of patients without traditional risk factors are constantly being discovered. Examples include patients after solid organ transplantation (SOT); patients with liver or kidney disease, chronic obstructive pulmonary disease (COPD), malnutrition or diabetes mellitus; critically ill patients with sepsis; and patients taking corticosteroids (particularly with doses of > 20 mg of prednisone or equivalent daily)^5^. Past outbreaks with influenza and severe acute respiratory syndrome coronavirus 2 (SARS-CoV-2) have also been associated with increased invasive *Aspergillus* infections with the lung as the mainly affected organ, termed virus associated pulmonary aspergillosis (VAPA). The outcome of invasive pulmonary aspergillosis (IPA) in ICU patients is grim: mortality rates are often > 50%^5–7^.

The true incidence of IPA is difficult to establish because a definite diagnosis requires a lung biopsy with histopathological examination showing invasive growth. In critically ill patients with respiratory failure, an impaired coagulation status and hemodynamic instability, performing a lung biopsy might be too risky. Therefore, non-biopsy-guided diagnostic algorithms have been established, integrating clinical, radiological and microbiological features. One of these algorithms by EORTC/MSG was revised in 2008^4^, but it was only applicable for patients under immunosuppression and/or with classical host factors. In 2012, Blot et al.^8^ reported the AspICU algorithm based on an external validation study incorporating ICU patients without traditional “host factors”. The AspICU study showed that the classical features on computed tomography (CT) scans, including the halo sign or the air-crescent sign, are rarely noted in patients without neutropenia. In these patients, an entry criterion to diagnose IPA in the AspICU algorithm is a positive lower respiratory tract specimen culture. Because researchers have described ≤ 65% sensitivity for lower respiratory tract specimen cultures after broncho-alveolar lavage (BAL)^9^, non-culture-based methods are urgently needed.

Galactomannan (GM) is a polysaccharide and a major component of the cell wall of *Aspergillus* spp^10^. Angioinvasion by *Aspergillus* spp. leads to the release of GM in the circulation^11^ and can be detected in different fluids (broncho-alveolar lavage fluid [BALF], serum and cerebrospinal fluid). BALF samples are superior to blood samples, especially in patients without neutropenia. In this population, a galactomannan index (GMI) in BALF reported as an optical density index (ODI) ≥ 1.0 is probably associated with an invasive disease. False-positive GM results have been described in a variety of situations such as *Histoplasma* infections^10^, therapy with intravenous human immunoglobulin^12^ or caspofungin administration^13^. A few case reports have indicated false-positive GM testing in patients after aspiration of enteral nutrition^14^ and in patients with aspiration pneumonia^15^. In another case, a patient with mucositis after HSCT had false-positive GM serum results caused by oral nutritional supplements^16^. Here, we examined the impact of false-positive GM results in BALF in a heterogeneous group of surgical patients who were specifically evaluated for the impact of aspiration.

## Results

### Baseline characteristics and demographics

We included a total of 160 patients in the study. Ninety-one patients (56.9%) were classified as non-IPA (colonisation only); we did not include these patients in the final analysis. In the remaining patients, 30 (18.8%) had pIPA and 39 (24.4%) had aspiration. The baseline characteristics of all included patients are displayed in detail in Table 1. Patients with aspiration were older compared with patients with pIPA (72 vs. 61 years, *p* < 0.01). Patients with pIPA showed a higher degree of illness: liver cirrhosis and SOT was more common in this group (50.0% vs. 5.1%, *p* < 0.001, and 63.0% vs. 0%, *p* < 0.001, respectively). Corticosteroid therapy was also significantly more common in patients with pIPA compared with patients with aspiration (97.0% vs. 21.0%, *p* < 0.001). The need for kidney replacement therapy was higher in patients with pIPA (40.0% vs. 7.7%, *p* = 0.001). The two groups also differed in terms of the ASA status (*p* = 0.04) and the SOFA scores at ICU admission (11 vs. 7 points, *p* < 0.01). The time spent in the ICU and the duration of mechanical ventilation was longer in patients with pIPA compared with patients with aspiration (47 vs. 28 days, *p* = 0.02 and 453 vs. 249 h, *p* = 0.07, respectively), indicating that patients with pIPA were more severely ill. In addition, the SOFA score in patients with pIPA was higher at the first detection of GM in BALF (13 vs. 11 points, *p* = 0.02). The first and highest GM values in BALF were significantly higher in patients with pIPA compared with patients with aspiration (5.8 vs. 2.7, *p* < 0.001 and 4.5 vs. 2.0, *p* < 0.01, respectively). *Aspergillus fumigatus* cultures from BALF were positive for 47.0% of patients with pIPA but only for 2.6% of patients with aspiration (*p* < 0.001). The patient groups differed significantly in terms of antifungal therapy; patients with pIPA received antifungal prophylaxis more often (23.0% vs. 5.1%, *p* = 0.04), a first-line antifungal therapy (90.0% vs. 38.0%, *p* < 0.001) and a switch to a second-line antifungal treatment (50.0% vs. 7.7%, *p* < 0.001) compared with patients with aspiration. Table 2 shows the microbiological test results and the corresponding antifungal treatments in all included patients.

**Table 1:** Baseline characteristics of all included patients.

*N* = 69	ASP (*n* = 39)	pIPA (*n* = 30)	*p*
Demographics			
Age [Years] †	72 (61, 76)	61 (54, 69)	< 0.01
Females	9 (23%)	9 (30%)	0.5
Underlying diseases			
COPD	7 (18%)	6 (20%)	0.8
Coronary artery disease	10 (26%)	10 (26%)	0.12
Diabetes mellitus	5 (13%)	7 (23%)	0.3
Heart insufficiency	12 (31%)	15 (50%)	0.10
Liver cirrhosis	2 (5.1%)	15 (50%)	< 0.001
Alcohol abuse	5 (13%)	6 (20%)	0.5
Chemotherapy	2 (5.1%)	3 (10%)	0.6
Solid organ transplantation	0 (0%)	19 (63%)	< 0.001
Liver	0 (0%)	13 (43%)	< 0.001
Kidney	0 (0%)	6 (20%)	< 0.01
Corticosteroid therapy	8 (21%)	29 (97%)	< 0.001
Because of sepsis	7 (18%)	10 (33%)	0.14
Bacteremia	10 (26%)	10 (33%)	0.5
Dialysis	3 (7.7%)	12 (40%)	< 0.01
Delirium	20 (59%)	14 (48%)	0.4
Stroke	6 (15%)	3 (10%)	0.7
ASA Status‡			0.04
2	7 (18%)	0 (0%)	
3	22 (56%)	17 (57%)	
4	9 (23%)	12 (40%)	
5	1 (2.6%)	1 (3.3%)	
SOFA ICU admission†	7 (3, 11)	11 (6, 16)	< 0.01
Time on ICU [d] †	28 (20, 45)	47 (28, 85)	0.02
Mechanical ventilation [h] †	249 (96, 589)	453 (187, 828)	0.07
Death at 28 days	12 (32%)	9 (31%)	0.9

Data are presented as n (%).

†Values are presented as median, (Interquartile range).

‡Values are presented as mean ± standard deviation.

*COPD* (Chronic obstructive pulmonary disease), *ASA* (American society of Anaesthesiology), *SOFA* (Sequential organ failure assessment), *ICU* (Intensive care unit).

**Table 2:** Microbiological tests and antifungal treatments in all patients.

*N* = 69	ASP (*n* = 39)	pIPA (*n* = 30)	*p*
SOFA first GM positivity†	11 (7, 13)	13 (10, 15)	0.02
BALF-positive GM			
First value of GM (BALF) †	2.0 (1.25, 4.32)	4.50 (2.28, 5.74)	< 0.01
Highest value of GM (BALF) †	2.7 (1.4, 4.9)	5.8 (4.5, 7.3)	< 0.001
*Aspergillus* spp. culture positive	1 (2.6%)	14 (47%)	< 0.001
Antifungal therapy			
Antifungal prophylaxis	2 (5.1%)	7 (23%)	0.04
First-line therapy [yes]	15 (38%)	27 (90%)	< 0.001
Second-line therapy [yes]	3 (7.7%)	15 (50%)	< 0.001

Data are presented as n (%).

†Values are presented as median, (Interquartile range).

SOFA (Sequential organ failure assessment), GM (Galactomannan), BALF (Broncho-alveolar lavage fluid).

### Outcome

The primary outcome, mortality within 28 days after ICU admission, did not differ between patients with pIPA and aspiration (31% vs. 32%, *p* = 0.9).

### Uni- and multivariate analyses

Only an elevated SOFA score (an increase of 1 point) during first GM positivity was associated with an increased 28-day mortality risk (HR 1.20, 95% confidence interval [CI] 1.04–1.39) in the multivariate analysis. By contrast, neither the highest GM value nor the SOFA score upon ICU admission were risk factors for 28-day mortality (*p* = 0.14 and *p* = 0.50, respectively). Table 3 lists the risk factors for death within 28 days after ICU admission.

**Table 3:** Risk factors for death within 28 days after ICU admission.

	Hazard ratio (95% CI)	*p*
Univariate analysis		
SOFA ICU admission	0.98 (0.9–1.1)	0.67
GM-SOFA first GM positivity	1.2 (1-1.3)	0.011
First value of GM (BALF)	0.96 (0.79–1.2)	0.71
Highest value of GM (BALF)	0.92 (0.77–1.1)	0.38
*Aspergillus spp.* culture positivity	1.2 (0.39–3.5)	0.78
Bacteremia	3.1 (1.3–7.4)	0.01
Aspiration	1.1 (0.43–2.9)	0.83
Parenteral nutrition	0.84 (0.25–2.9)	0.78
Enteral nutrition	1.4 (0.56–3.7)	0.45
Multivariate analysis		
SOFA ICU admission	0.96 (0.87–1.07)	0.50
GM-SOFA first GM positivity	1.20 (1.04–1.39)	0.01
No antifungal therapy	0.60 (0.17–2.06)	0.41
Highest value of GM (BALF)	0.83 (0.65–1.06)	0.14

### Probability of aspiration

**Fig. 1:**
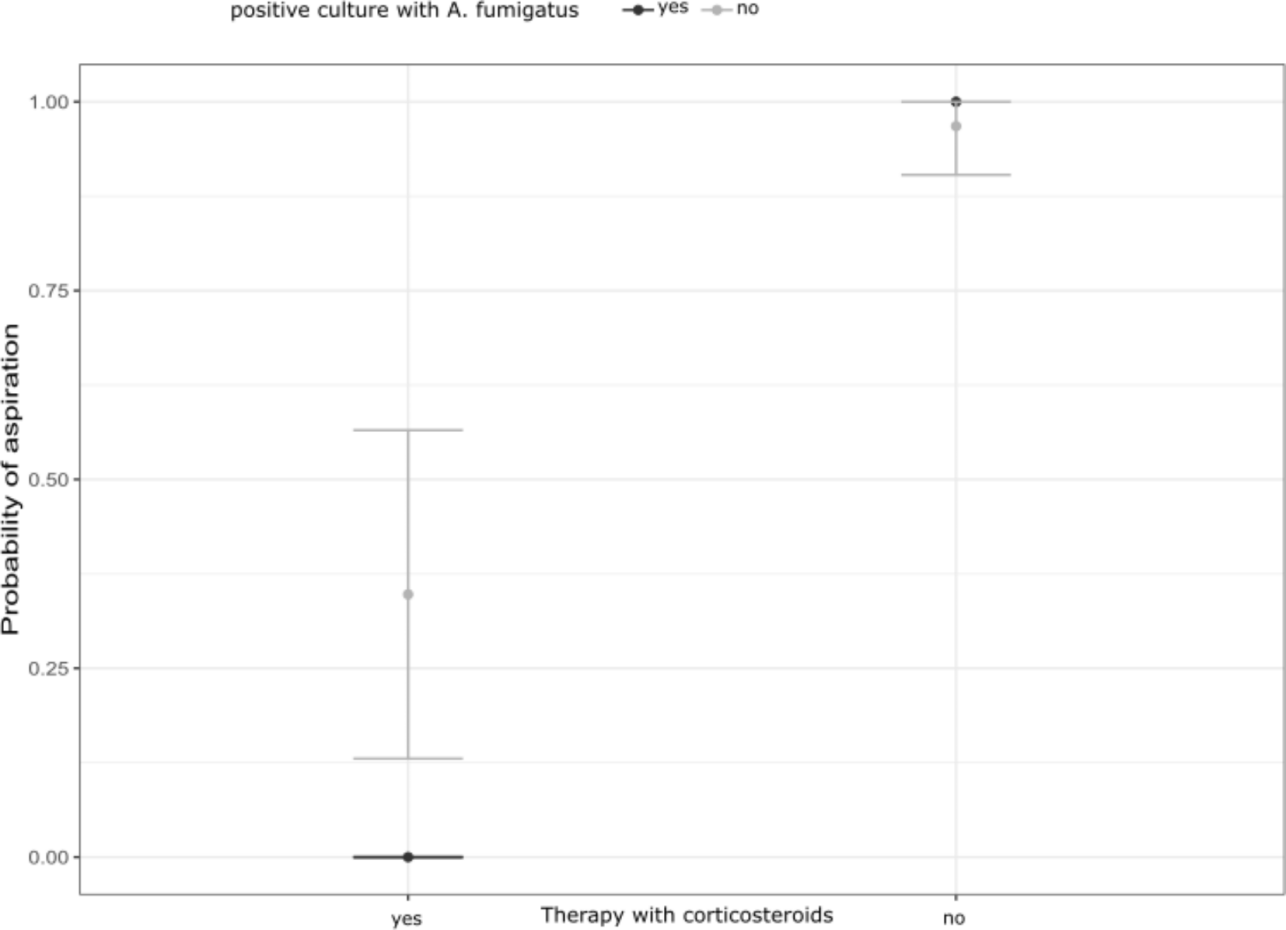
Logistic model predicting probability of aspiration: No therapy with corticosteroids and a negative *Aspergillus* spp. culture predicts a high probability of aspiration with a co-efficient of discrimination (Tjur’s R^2^) of 0.65.

We developed a logistic model including the use of corticosteroids and positivity of *Aspergillus* culture to predict the patient group membership. The model’s explanatory power was substantial with a co-efficient of discrimination (Tjur’s R^2^) of 0.65, and both effects were statistically significant: no usage of corticosteroids and a negative *Aspergillus* culture predict a high probability of belonging to the aspiration group (Fig. 1). We also calculated sensitivity and specificity for the probability of belonging to the pIPA group in our patient cohort. Interestingly, a combination of a first GM value in BALF and a positive *A. fumigatus* specimen culture from BALF had a sensitivity of 0.5 and a specificity of 0.86. The combination of the highest GM value in BALF and a positive *A. fumigatus* specimen culture from BALF showed a higher sensitivity (0.7) but a lower specificity (0.8). These findings again point towards the fact that a negative *Aspergillus* specimen culture can rule out pIPA in patients with a low-risk profile or signs of aspiration.

### Enteral nutritional supplementation

We tested several enteral nutritional supplements with the Platelia™ Aspergillus ELISA in our department. Most striking was the fact that the higher the amount of soy in the supplements, the higher the measured GM values. In fact, every supplement tested positive for GM, but the lower the fraction of protein (and the less soy-based protein), the lower the GM values. Table S2 includes the values for the enteral nutritional supplements we tested. No difference was seen in terms of application of parenteral- or enteral nutrition between pIPA and ASP-patients in the last three days before GM detection in BALF (93% vs. 77%, *p* = 0.2 and 70% vs. 59%, *p* = 0.6 respectively). Additional information about the distribution of each supplement in each group is provided in Table S3.

## Discussion

Here, we have described the prevalence and outcome of patients with positive GM detection in BALF due to aspiration or pIPA in a mixed cohort of surgical ICU patients. IPA is most often diagnosed in patients with “classical” risk factors (e.g. neutropenia, haematological malignancy and recipients of an allogeneic HSCT)^4,10,17^. The group of at-risk patients with non-traditional risk factors (e.g. SOT recipients, sepsis, severe viral infections, diabetes mellitus, COPD, malnutrition and liver cirrhosis) has increased steadily over the last 10–20 years^18–21^. The AspICU study^8^, an international, multicentre study examining the incidence of IPA in 30 ICUs in 8 countries, showed that > 90% of included patients had co-morbidities: respiratory diseases (including COPD), cardiovascular disease, diabetes mellitus and immunosuppressive therapy. We found similar results in our cohort. Most patients with pIPA presented with additional risk factors: >50% of patients were SOT recipients, 50% had heart insufficiency and 33% received corticosteroids because of sepsis. Even though some patients with pIPA lack traditional risk factors, they are usually more severely ill. For example, the SOFA scores in the AspICU cohort^8^ were higher in proven IPA (median 11, interquartile range [IQR] 7–14) or pIPA (median 9, IQR 6–12) compared with patients with *Aspergillus* colonisation only (median 5, IQR 2–9). In our study, patients with pIPA also showed significantly higher SOFA scores upon ICU admission (11 vs. 7 points) and during the first GM positivity (13 vs. 11 points) compared with patients with aspiration. This is in line with our last published study^22^, in which we also showed that patients with pIPA presented with higher SOFA scores upon ICU admission compared with patients with *Aspergillus* colonisation only (11 vs. 8 points, *p* = 0.04). In a retrospective study with patients with severe COVID-19 and concomitant COVID-19-associated pulmonary aspergillosis (CAPA), elevated disease severity (as assessed by the SOFA score) and the need for organ support were also significantly linked to a worse outcome^23^.

In a multicentre study based in Italy, previous use of corticosteroids, mainly due to autoimmune disease or COPD, was a major risk factor in developing IPA^24^. IPA risk is also augmented in patients receiving corticosteroids only during their hospital stay. This cohort of patients is increasing, especially those admitted to the ICU with septic shock and requiring high doses of vasopressors^11^. In our cohort, 97% of patients with pIPA received corticosteroid therapy, mostly due to SOT or sepsis. Corticosteroid therapy increases the susceptibility to opportunistic infections, including invasive aspergillosis^25^. This is primarily through its immunomodulatory effects on immune cells. For example, glucocorticoids reduce the production of reactive oxygen species (ROS) and LC3-associated phagocytosis (LAP), leading to an altered fungicidal ability of myeloid cells^26^. In a human ex vivo cell model with neutrophil granulocytes from healthy volunteers and *A. fumigatus*, the *Aspergillus* defence provided by neutrophil granulocytes was significantly stronger in the afternoon than in the morning, due to potentially (significantly) different cortisol levels measured in the included volunteers^27^. A systematic review and meta-analysis of patients with severe influenza clearly showed a significant relation between corticosteroid use and IPA^6^. Data regarding other underlying diseases, such as COVID-19, are more conflicting. A recently published meta-analysis with more than 6000 patients with COVID-19 indicated that treatment with corticosteroids is a significant risk factor for developing CAPA^28^, although the actual guidelines recommend 6 mg of dexamethasone per day, a potent glucocorticoid, for up to 10 days, in patients with severe COVID-19^29^. Corticosteroids should be prescribed with caution in critically ill patients, and physicians must weigh the risks against the benefits, especially in patients with a high risk for IPA.

Although the Platelia™ assay was initially only approved to measure GM in serum, BALF was added as a validated sample type in 2011^10^. There is still a debate about the cutoff that should be used. With a GM cutoff of ≥ 1.0 in BALF, as proposed in the updated consensus definitions by EORTC/MSGERC in 2021^10^, sensitivity ranges from 0.75 to 0.86 and specificity ranges from 0.94 to 0.95. Compared with GM in serum, the sensitivity of GM in BALF is similar in patients with or without haematological diseases and in patients with and without neutropenia.

The outcomes in our study did not differ between patients with pIPA and aspiration. This is most likely due to the timely and accurate diagnostic workup of patients with pIPA rather than IPA itself. Another reason might lie in the severe illness of all included patients. The SOFA scores during first GM positivity were > 10 points in all patients. Only an elevated SOFA score (an increase of 1 point) during first GM positivity was independently linked to an increased 28-day mortality risk.

As with false positive GM in serum^30^ (e.g. mucositis in patients after autologous hematopoietic stem cell transplantation) there are numerous possibilities of false positive GM in BALF, like infections with closely related fungi^10^ (e.g. *Histoplasma capsulatum*, *Fusarium* spp., *Cryptococcus* spp. and *Penicillium* spp.) or aspiration of enteral nutrition supplements^15^. To our knowledge, no study has systemically studied the impact of aspiration in critically ill patients as a potential source of false-positive GM in BALF.

Dysphagia is a well-known risk factor for aspiration and leads to an increased morbidity and mortality in ICU patients^31^. Depending on the patients screened and diagnostic tool used, up to 30% of ICU patients are diagnosed with dysphagia^31,32^. Higher age, tube feeding, sepsis and duration of mechanical ventilation are known risk factors for dysphagia^32^. In our own cohort, patients with aspiration were all mechanically ventilated over 10 days (a median of 249 h) and presented with an older age (> 10 years older than patients with pIPA), which are both common risk factor for dysphagia and aspiration^33^. In the NUTRIREA-2 trial^34^, the authors explored the impact of nutrition route (enteral vs. parenteral) on microaspiration in critically ill patients with shock in a mixed cohort of patients. Results showed that both ways of nutritional support led to high rates of microaspiration, although vomiting was significantly more common in the enteral feeding group (31% vs. 15%, *p* = 0.016). Because almost 90% of the included patients were admitted to the ICU because of a medical reason and only around 10% were admitted due to planned or urgent surgery, these results are not comparable to our study. Our cohort only included surgical patients. Most of these patients received both parenteral and enteral feeding simultaneously (data not shown). Therefore, it is not surprising that 24% of the patients included in our study showed signs of aspiration (and false-positive GM in BALF). The term “aspiration” is not clearly defined in the literature and no single theory has been accepted so far since it lacks clear definitions. The definition proposed in this manuscript by Mandell et al.^35^ is not the only definition but one that incorporates pathogenetic and predisposing factors and hence seemed to be the best available option. A recent review published by the Japanese Study Group on Aspiration Pulmonary Disease^36^ proposed clinical diagnostic criteria. This algorithm differed between “certain cases” (direct observation of aspiration) and “probable or suspected cases”, where swallowing function disorders were apparent in the latter diagnosis. This is very close to the diagnostic criteria used in our study.

Because we suspected the enteral nutritional supplements were responsible for the false-positive GM results, we tested several enteral supplements from different companies. Most interestingly, supplements with a high soy-based ratio showed higher GM values. Moreover, the GM values were slightly elevated in milk-protein-based supplements without soy. One reason might be that *Aspergillus* spp. were used in the fermentation process^37^. Galactomannan often contaminates the final solutions even after the filtration process^10^. Ansorg et al. already published over 20 years ago^38^ that vegetables, cereals and fruit contain components that react in the latex agglutination test The authors assumed that because of the specificity of the monoclonal antibody of the test, the components are probably GM and contamination. In recent reports, researchers have assumed that these contaminants enter the bloodstream through a disrupted gastrointestinal barrier (mucositis or graft-versus-host disease), resulting in false-positive GM serum test results. In our study, we did not directly test the BALF for the presence of nutritional components but assumed that some presence of nutritional supplements should have been the cause of false- positive GM BALF test results.

Only 1 of the 39 patients with aspiration showed a positive culture specimen for *Aspergillus* spp., even though GM in BALF was as high as 1.98 (IQR 1.25–4.32) and hence significantly higher than proposed by actual guidelines for GM cutoffs in BALF^10^. This finding supports our assumption that these patients presented with false-positive GM BALF test results. Nevertheless, 38% of patients with aspiration may have received an unnecessary antifungal therapy. This is worrisome because antifungal therapies with triazoles carry potential side effects in critically ill patients, like severe drug-drug interactions, dose-adjustment in severe kidney and liver failure, neurologic disturbances and QT prolongation^39^.

In summary, one can say that if there is clear evidence of aspiration, elevated GM values (independent of their maximum values) may not be linked to IPA. This is especially true in cases with missing risk factors such as glucocorticoid therapy. In our model, no use of corticosteroids together with a negative *Aspergillus* culture predicted a high probability of aspiration (Tjur’s R^2^ = 0.65). Nonetheless, even a positive *Aspergillus* culture specimen from BALF does not automatically reflect invasive *Aspergillus* growth, especially in the absence of abnormal medical imaging or appropriate signs and symptoms^19^.

Aspiration in patients with enteral nutritional supplements seems to be an important issue as a potential cause of false-positive GM BALF test results. Nevertheless, positive GM values (and other mycologic evidence of *Aspergillus* spp.) in BALF need to be seriously considered in critically ill patients. Interpretation should always involve the patient risk factors, including medical history underlying conditions and mycological results^19^.

**Fig. 2:**
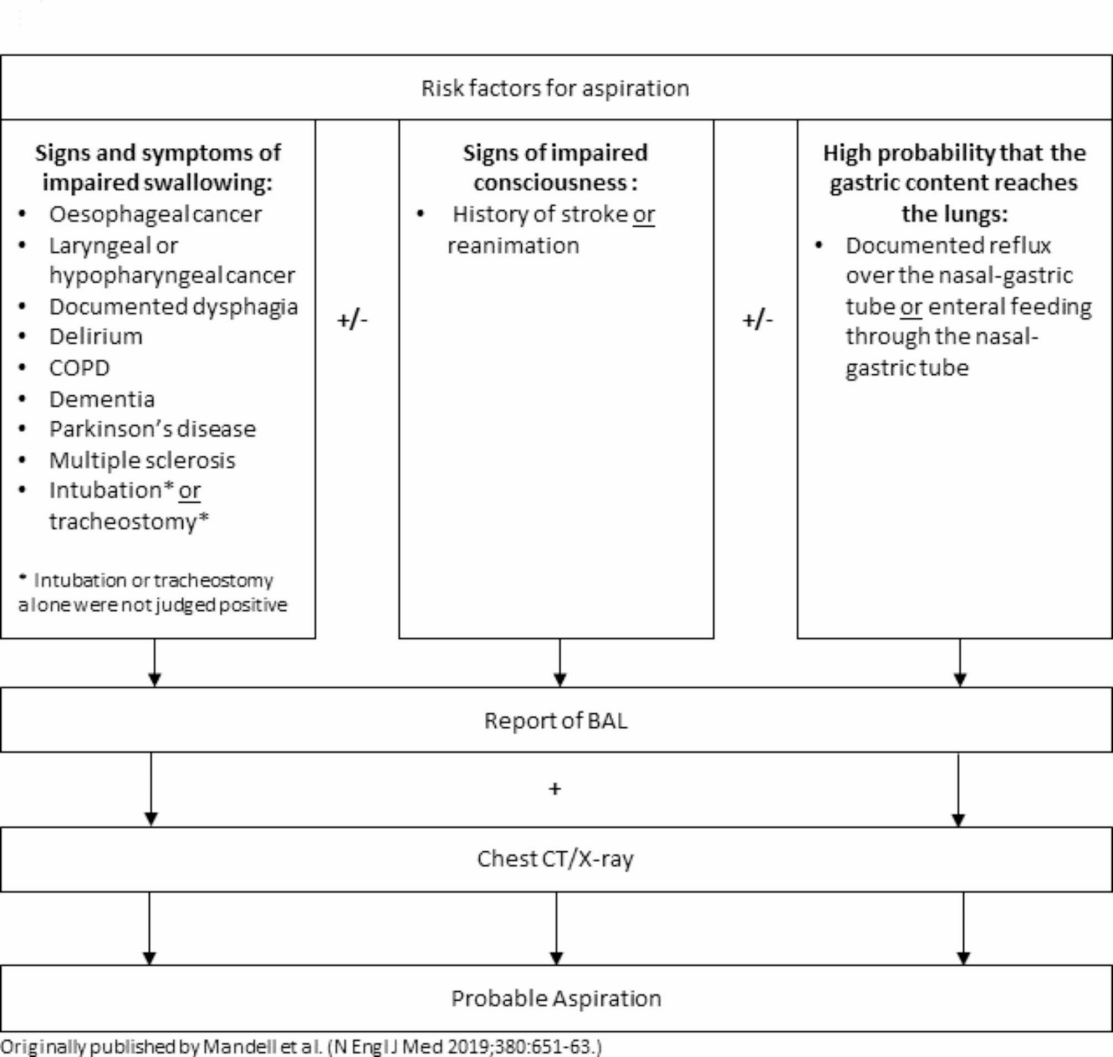
Diagnostic algorithm for the diagnosis of probable aspiration. Abbreviations: COPD (chronic obstructive pulmonary disease), BAL (Broncho-alveolar-lavage), CT (computed tomography).

### Ethical statement

*Statement of human rights*: All procedures performed in studies involving human participants were done in accordance with the ethical standards of the institutional research committee [approval by the institutional review board of the medical faculty of the University of Heidelberg, Germany (S-191/2018)] and with the declaration of Helsinki and its later amendments or comparable ethical standards.

*Informed consent*: The approval committee has waived informed consent.

## Methods

### Study design

This study was conducted retrospectively at a single surgical ICU at Heidelberg University Hospital, Heidelberg (Germany). Data were extracted from patients who were hospitalised between March 2014 and December 2019. Heidelberg University Hospital is a regional reference centre for SOT (especially kidney and liver). Informed consent was deemed unnecessary according to national regulations and due to the retrospective nature of the study. The presented protocol was approved by Institutional Review Board (IRB) (Ethics committee of the medical faculty, Heidelberg university hospital, Heidelberg, Germany: S-191/2018). In addition, the study was registered at the German Clinical Trials Register (DRKS-ID: DRKS00024735) as a secondary analysis of data published in 2023^22^.

### Patient population

Only adult (≥ 18 years) surgical ICU patients with at least one BALF sample with a GM ODI ≥ 1.0 according to the manufacturer’s instructions (Platelia™ *Aspergillus* enzyme-linked immunosorbent assay [ELISA], Bio-Rad, Marnes-la-Coquette, France) and one CT scan of the chest or chest X-ray were included in the study.

### Patient data

Data were collected from an electronic medical record system (ISH^®^, SAP, Walldorf, Germany). The following items were extracted: underlying diseases (COPD, coronary artery disease, diabetes mellitus, heart insufficiency, liver cirrhosis, solid tumour, alcohol abuse, SOT, chemotherapy and history of stroke) and complications during the ICU stay (bacteraemia, need for dialysis, corticosteroid therapy and duration of mechanical ventilation). The American Society of Anesthesiologists (ASA) classification and the Sequential Organ Failure Assessment (SOFA) score were collected to assess disease severity.

### Outcomes

The primary endpoint was 28-day mortality (from any cause) after ICU admission. This endpoint was chosen due to fact, that major epidemiologic outcome studies in critical care patients chose the 28-day (or 30-day) mortality endpoint^1,40^. This makes our results comparable to other outcome studies in the intensive care setting.

### Definitions

#### (p)IPA

IPA was defined according to the AspICU criteria (Table S1). According to Schröder et al.^41^ in addition to AspICU criteria, a GM ODI ≥ 1.0 in BALF samples also served as an entry criterion for IPA diagnosis. Patients fulfilling all four criteria (clinical data + radiological findings + host factors + mycological finding) were termed putative IPA (pIPA).

#### Aspiration

Aspiration was defined when either “definitive” or “probable” aspiration occurred during an ICU stay, triggering a bronchoscopy from the responsible staff. “Definitively aspirated” was defined as vomiting and/or documented aspiration. “Probably aspirated” (Fig. 2) was defined based on anamnesis, reported of BAL and chest CT/X-ray and documented risk factors for aspiration according to Mandell et al.^35^: signs and symptoms of impaired swallowing (documentation of impaired swallowing, operation due to oesophageal cancer, delirium, COPD, dementia, Parkinson’s disease, multiple sclerosis, intubation and tracheostoma); signs of impaired consciousness (history of stroke or reanimation); and a high probability that the gastric content reaches the lungs (documented reflux over the nasal-gastric tube or enteral feeding through the nasal-gastric tube).

#### Second-line antifungal therapy

The decision to add a new antifungal agent or to switch to another antifungal drug was driven by mycological evidence (e.g. direct [cultured] or indirect [GM] evidence of *Aspergillus* spp.) in BALF, radiological evidence (e.g. ongoing radiological evidence of IPA on chest CT/X-ray) or clinical evidence (e.g. a decrease in the respiratory status despite ongoing antifungal therapy).

#### Corticosteroid therapy

Corticosteroid therapy was defined as the use of prednisolone > 20 mg/day or another corticosteroid at an equivalent dosage during or before the hospital stay prior to the first GM detection in BALF.

#### Microbiology

*Aspergillus* spp. isolates, grown from respiratory specimen isolates, were investigated at the Department of Medical Microbiology and Hygiene, Bacteriology Division of University Hospital Heidelberg, Heidelberg, Germany. Only positive cultures with *Aspergillus* spp. were included in the study. GM testing was performed with the Platelia™ *Aspergillus* ELISA (Bio-Rad). Microbial growth in blood culture bottles was detected as described by Dubler et al.^22^.

#### BAL

BAL for invasively ventilated or awake patients was performed according to a local standardised protocol, which has been described in detail in a previous study^22^.

### Statistics

The data was stored in Excel (Microsoft^®^, Redmond, WA, USA) and then analysed in R (cite with: R Core Team (2022). R: A language and environment for statistical computing. R Foundation for Statistical Computing, Vienna, Austria. URL https://www.R-project.org/.) using the packages survminer (Alboukadel Kassambara, Marcin Kosinski and Przemyslaw Biecek (2021). survminer: Drawing Survival Curves using ‘ggplot2’. R package version 0.4.9. https://CRAN.R-project.org/package=survminer) and survival (Therneau T (2021). _A Package for Survival Analysis in R_. R package version 3.2–13, < URL: https://CRAN.R-project.org/package=survival>.) for survival analysis and gtsummary (10.32614/RJ-2021-053) to display the data in tables. Continuous data are presented as the mean and standard deviation. Categorical variables are displayed as absolute and relative frequencies. The Mann–Whitney U-test or the chi-square test was used to calculate potential differences between the groups. Kaplan–Meier curves present survival information. Cox proportional hazards regression model with adjustment for potential confounders was used to identify risk factors for mortality (hazard ratio [HR]). Two-sided *p* < 0.05 was considered statistically significant for all analyses.

## Electronic supplementary material

Below is the link to the electronic supplementary material.

Supplementary Material 1

## Data Availability

Data are available only on request. Please use the following contact information from the author: simon.dubler@uk-essen.de.
