# Supplementary material for: Implications for the diagnosis of aspiration and aspergillosis in critically ill patients with detection of galactomannan in broncho-alveolar lavage fluids
Source: Sci Rep. 2025 Jan 15;15:1997. doi: 10.1038/s41598-025-85644-5 (PMC11735627; doi:10.1038/s41598-025-85644-5)
Supplement: Supplementary file 1 — Supplementary Material 1 [file 41598_2025_85644_MOESM1_ESM.docx]

**Supplementary Material**

**Table S1: AspICU alternative clinical algorithm according to Blot et al (2012)**

| **Putative invasive pulmonary aspergillosis (all four criteria must be met)** |
| --- |
| 1. Aspergillus-positive lower respiratory tract specimen culture (entry criterion) |
| 2. Compatible signs and symptoms (one of the following) |
| - Fever refractory to at least 3 d of appropriate antibiotic therapy |
| - Recrudescent fever after a period of effervescence of at least 48 h while still on antibiotics and without other apparent cause |
| - Pleuritic chest pain |
| - Pleuritic rub |
| - Dyspnoea |
| - Hemoptysis |
| - Worsening respiratory insufficiency despite appropriate antibiotic therapy and ventilatory support |
| 3. Abnormal medical imaging by portable chest X-ray or CT scan of the lungs |
| 4. Either 4a or 4b |
| 4a. Host risk factors (one of the following conditions) |
| - Neutropenia (absolute neutrophil count ,500/mm3) preceding or at the time of ICU admission |
| - Underlying hematological or oncological malignancy treated with cytotoxic agents |
| - Glucocorticoid treatment (prednisone equivalent >20 mg/d) |
| - Congenital or acquired immunodeficiency |
| 4b. Semiquantitative *Aspergillus*-positive culture of BAL fluid (+ or ++), without bacterial growth together with a positive cytological smear showing branching hyphae |
| ***Aspergillus* respiratory tract colonization** |
| When >1 criterion necessary for a diagnosis of putative IPA is not met, the case is classified as *Aspergillus* colonization. |

Abbreviations: BAL (Bronchoalveolar lavage), CT (Computed tomography), EORTC/MSG (European Organization for the Research and Treatment of Cancer/Mycosis Study Group), ICU (Intensive care unit)

**Table S2: Galactomannan values of different enteral nutrition supplements**

|  | **Galactomannan-values in Platelia™ Aspergillus ELISA (in dilution rows)** | | | | **Soy-based?** | **% protein per 100ml** |
| --- | --- | --- | --- | --- | --- | --- |
| **Brand name** | **Pure** | **1:50** | **1:500** | **1:2500** | **If yes; % of whole** |  |
| Nutrison Soja Multi Fibre® | 5,91 | 4,87 | 3,06 | 0,60 | 100% | 16% |
| Hipp enteral nutrion ® (normocaloric) | 5,23 | 4,76 | 2,53 | 0,49 | 80% | 15% |
| Nutrison Protein Plus® | 5,24 | 5,29 | 2,30 | 0,78 | 20% | 20% |
| Fresubin Hepa® | 4,81 | 4,98 | 2,06 | 0,40 | 36% | 12% |
| Nestle Peptamen® | 5,15 | 3,06 | 1,26 | 0,42 | 0% | 16% |
| Restoric nephro intensiv® | 4,64 | 0,80 | 0,11 | 0,07 | 0% | 14% |
| Nestle Isosource® | 3,24 | 0,25 | 0,09 | 0,05 | 0% | 16% |
| Fresubin Energy Fibre® | 2,4 | 0,3 | 0,1 | 2,4 | 49% | 15% |
| Fresenius Survimed® | 1 | 0,1 | 0,1 | 1 | 0% | 20% |
| Fresubin Original Fibre® | 1,5 | 0,2 | 0,1 | 1,5 | 49% | 15% |
| Fresubin High protein Fibre® | 0,4 | 0,1 | 0,1 | 0,4 | 0% | 20% |
| Fresenius Diben High protein® | 0,9 | 0,1 | 0,1 | 0,9 | 0% | 20% |
| Fresubin Energy Fibre Drink (Caramel)® | 0,6 | 0,1 | 0,1 | 0,6 | 0% | 15% |
| Fresubin Renal Vanille Drink® | 0,4 | 0,3 | 0,1 | 0,4 | 0% | 6% |

**Table S3: Distribution of parenteral and enteral nutrition**

| **N=69** | **ASP (n=39)** | **pIPA (n=30)** | ***p*** |
| --- | --- | --- | --- |
| Parenteral nutrition (yes) | 30 (77%) | 28 (93%) | 0.2 |
| Enteral nutrition (yes) | 23 (59%) | 21 (70%) | 0.6 |
| Fresubin Hepa^©^ | 0 (0%) | 5 (17%) |  |
| Fresubin Energy Fibre^©^ | 1 (2.7%) | 0 (0%) |  |
| Isosource Optifibre^©^ | 14 (38%) | 11 (37%) |  |
| Nutrison Energy^©^ | 0 (0%) | 0 (0%) |  |
| Fortijuice^©^ | 0 (0%) | 0 (0%) |  |
| Peptamen^©^ | 3 (8.1%) | 0 (0%) |  |
| Fresubin HP Energy^©^ | 0 (0%) | 1 (3.3%) |  |
| Nutrison Multifibre^©^ | 0 (0%) | 0 (0%) |  |
| Nutrison Soya^©^ | 2 (5.4%) | 0 (0%) |  |
| Nutriflex Omega^©^ | 1 (2.7%) | 0 (0%) |  |
| Nepro HP^©^ | 1 (2.7%) | 3 (10%) |  |
| Restoric Nephro Intensiv^©^ | 0 (0%) | 1 (3.3%) |  |
| Unknown | 2 | 0 |  |
|  |  |  |  |
